# The influence of environmental factors on breeding system allocation at large spatial scales

**DOI:** 10.1093/aobpla/ply069

**Published:** 2018-11-08

**Authors:** Beth H Ansaldi, Steven J Franks, Jennifer J Weber

**Affiliations:** 1 Department of Biology, Fordham University, Bronx, NY, USA; 2 Department of Biology, Southeast Missouri State University, Cape Girardeau, USA

**Keywords:** Breeding systems, Campanulaceae, cleistogamy, geographic analyses, reproductive allocation

## Abstract

Plant breeding systems can vary widely among populations, yet few studies have investigated abiotic factors contributing to variation across a broad geographic range. Here we investigate variation in reproductive traits of *Triodanis perfoliata* (Campanulaceae), a species that exhibits dimorphic cleistogamy, a condition in which individual plants have both closed (selfing: cleistogamous: CL) and open (selfing or outcrossing: chasmogamous: CH) flowers. Chasmogamous production is theorized to be more costly because CH flowers have a larger exposed surface area and thus are more likely to lose more water than CL flowers. We examine relationships between abiotic conditions (temperature, precipitation and soil characteristics) and variation in breeding systems across 14 widespread populations using ordinary least squares models. We found that a large proportion of breeding system variation was described by climate and soil variables (*R*^2^ = 0.65–0.92). These results support the hypothesis that variation in the environment drives variation in breeding system allocation. Our broad geographic analyses provide a framework for mechanistic studies of cleistogamy, and employ a novel approach for examining reproductive traits and environmental variation at large scales. Given that two major components of our models were temperature and precipitation, our study further emphasizes the potential for ongoing climate change to alter plant breeding systems.

## Teaser

Few studies have investigated how abiotic factors correlate with variation in plant breeding systems across broad geographic ranges. *Triodanis perfoliata* is a species with considerable variation in reproductive traits, including the relative production of two different types of flowers. Breeding system variation was accurately modelled with climate and soil variables across 14 geographically widespread populations of this species. This study uses a novel approach to examine how variation in the environment can drive variation in breeding system allocation at large spatial scales. Our study further emphasizes the potential for ongoing climate change to alter plant breeding systems.

## Introduction

The role of environmental factors in driving phenotypic trait variation among populations is a central focus in ecology and evolutionary biology. Such phenotypic variation includes variation in plant breeding systems, which are remarkably diverse (Barrett 2002). Although breeding system variation could potentially be determined or influenced by environmental conditions, few previous studies have examined associations between abiotic factors and reproductive traits beyond a handful of study populations. This is the case despite the fact that climate change has been predicted to alter plant breeding systems (Jones *et al.* 2013; Petry *et al.* 2016), but this would occur only if there is a link between abiotic conditions and breeding system variation. One reason that such a link has not been firmly established is that studies of breeding system trait variation often focus on a relatively small spatial scale and a few populations, meaning that abiotic variation is limited. In this study, we use a large spatial scale to examine how abiotic factors can influence plant breeding systems among 14 populations.

Over 650 species widely distributed across flowering plants exhibit cleistogamy, a breeding system in which at least some proportion of flowers on each individual lack an open corolla and are obligately selfing (cleistogamous (CL) flowers; Culley and Klooster 2007). Of these species, most exhibit dimorphic cleistogamy (Culley and Klooster 2007), and also produce hermaphroditic, facultative outcrossing flowers, called chasmogamous (CH) flowers. Individuals of species with dimorphic cleistogamy can vary in the relative proportion of CL and CH flowers (Lord 1981) with the relative allocation to different floral types likely dictated by both genetic and environmental factors. Plasticity in allocation to cleistogamy is considered a primary advantage of this breeding system (Schoen and Lloyd 1984).

Evolutionary theory suggests that stochastic environmental factors may favour dimorphic cleistogamy by oscillating between CH-favourable and CL-favourable conditions (Schoen and Lloyd 1984; Schoen and Brown 1991). For example, when pollinators are abundant, CH provides the opportunity for outcrossing and subsequently, and avoidance of inbreeding depression (Charlesworth and Charlesworth 1987). Though in many CL species, levels of inbreeding depression are relatively low, so this may not actually explain the maintenance of CH in many species (reviewed in: Oakley *et al.* 2007). Abiotic factors are also expected to have a strong influence on CL breeding systems due to the cost disparity between CL and CH reproduction. In CL flowers, smaller floral parts (reduced androecium, absence of corolla and nectar; Lord 1981) lower the energetic expense of seed production, compared to CH seed production (Oakley *et al.* 2007). Schoen and Lloyd (1984) modelled how different scenarios (e.g. levels of inbreeding depression, floral costs) favour CH, CL or a mixture of both floral types under various scenarios in perennials. Based on these models, both floral types are only favoured in a single season when the success of offspring from CL or CH varies based on the environment of the maternal plants. But in multiple scenarios only CL floral production is favoured in a single season, a pattern supported in some perennial species (e.g. Winn and Moriuchi 2009). Not only have far fewer studies examined allocation to CH or CL in annual species, but overall the conditions by which CH would be maintained evolutionarily remain an evolutionary mystery.

Consistent with the Schoen and Lloyd (1984), empirical work has illustrated that variation in allocation to cleistogamy may be associated with variation in environmental conditions (Schemske 1978; Clay 1983; Cheplick 2007). These studies and others generally support the hypotheses that plant breeding systems can be plastic and influenced by environmental conditions, but most studies of cleistogamy cover a limited geographic range. Currently, we have large gaps in our understanding about the extent of reproductive variation among populations of CL species and abiotic drivers of that variation (Culley and Klooster 2007). Our current study expands upon work in the annual *Triodanis perfoliata* (Campanulaceae), which has shown variation in the extent of cleistogamy among populations in North Carolina (Goodwillie and Stewart 2013; Colbert 2015). Previous work demonstrated that artificially manipulated light and soil condition can influence the breeding system, with increased light increasing the proportion of CH flowers produced (Colbert 2015).

**Figure 1.**
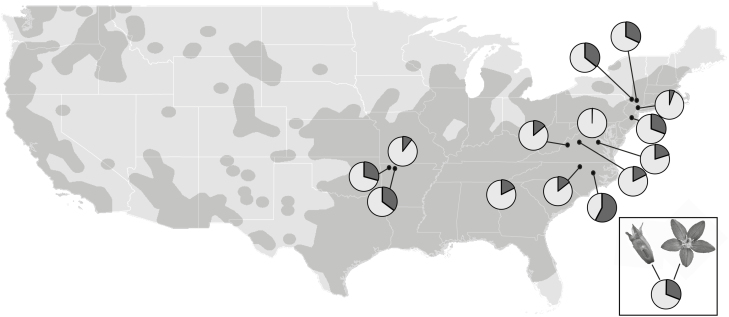
The overall range of *Triodanis perfoliata* across the contiguous USA based on vetted data from GBIF (GBIF 2018) shown in dark shading; light lines indicate the borders of states. The approximate location of the 14 study populations are indicated by black dots; pie charts represent the relative proportions of floral types in each population: dark grey = chasmogamous, white = cleistogamous.

If variation in cleistogamy is influenced by environmental factors (e.g. climate, soil composition), then we would expect to see variation across populations that occur in a wide range of environmental conditions. Overall, we hypothesize that environmental factors measuring potentially limiting resources (e.g. water availability) may correlate to CH production, with wetter areas producing more CH. Moreover, some combination of these spatially explicit environmental factors should accurately predict breeding system variation. In this study, we used a broad-scale sampling approach (spanning 1769 km) across 14 populations to examine the relationship between long-term abiotic conditions (temperature, precipitation and soil characteristics) and breeding system variation in *T. perfoliata* (Campanulaceae; Fig. 1). Ordinary least squares (OLS) models, which choose the parameters of a linear function, were used to identify environmental predictors of breeding system variation. Breeding system traits used in this study include total flower production, flower production by flower type and relative production of CH flowers. This study represents one of the first large-scale studies of inter-population variation in CL breeding systems.

## Materials and Methods

### Study species

*Triodanis perfoliata* (Campanulaceae) is a dimorphic CL ruderal annual herb. Native to North America, *T. perfoliata* typically grows in fields and areas subject to frequent disturbance such as roadsides, fields and pathways (Goodwillie and Stewart 2013). Flowering phenology varies across the USA, but generally begins from May to July and lasts several weeks (Goodwillie and Stewart 2013). The breeding system of *T. perfoliata* follows a typical spatial and temporal inflorescence pattern of an annual CL species: basal CL flowers occur first, followed by concurrent production of CH and secondary CL flowers, and ending in tertiary CL flower production. Each CH flower has an apparent bluish violet corolla, measuring 1–1.5 cm in width, whereas CL flowers lack a corolla. The mature capsules of *Triodanis* remain on the stem through senescence and seeds disperse from a small pore in the side of the fruit. The capsule of each flower type is also distinct; CL flowers most often have 3–4 sepals compared to five sepals on CH flowers (Trent 1940). These morphological distinctions, paired with typical differences in the ordinal arrangement along the stem, allow for accurate identification of each flower type on senesced plants.

#### Sampling plant reproductive traits.

In the summer of 2014, 14 populations were sampled that spanned four USDA plant hardiness zones (areas that encompass a certain range of climatic conditions for plant growth; United States Department of Agriculture 2018), we estimated range limits using vetted occurrence data from GBIF (Global Biodiversity Information Facility 2018) (Fig. 1). We estimated the total average production of each flower type in each population by collecting individual whole, senesced plants at the end of the growing season (range = 13–100; 49 = mean individuals per population). Breeding system traits were defined as the number of CH flowers (CH#), number of CL flowers (CL#), total flowers (TF) and proportion of flowers that were CH out of the total flower number (pCH).

### Mixed models of breeding system variation

The goal of the mixed spatial analyses was to determine the combination of climatic and soil environmental layers that best predict the observed breeding system traits in our 14 study populations. We examined potential correlations between breeding system traits and 19 standard bioclimatic variables provided by Worldclim 1.4 that reflect 30-year averages (Fick and Hijmans 2017) and 18 soil characteristics provided by ISRIC-WDC Soils (Hengl *et al.* 2014; Table 1). All plant variables were assessed for normality using Shapiro–Wilk tests, and for homogeneity of variances using Levene’s tests (Sokal and Rohlf 2012). Deviations from normality resulted in square-root transformation of CH flower number data.

**Table 1.** The potential climatic and soil parameters for building our models prior to reducing multicollinearity, parameters provided by Worldclim 1.4 (Fick and Hijmans 2017) and ISRIC-WDC Soils (Hengl *et al.* 2014), respectively. Soil parameters were available at two sampling depths: 10 and 150 cm and these data were highly correlated for each parameter. Parameters included in our models were reduced to avoid multicollinearity; the parameters included in our models are shown in bold. All soil parameters included in our models were measured at 10 cm sampling depth.

	Climatic parameter		Soil parameter
Bio_1	Annual mean temperature	BLD	Bulk sampling depth
**Bio_2**	**Mean diurnal range (mean of monthly (max temp–min temp))**	CEC	Cation exchange capacity
Bio_3	Isothermality	**CLYPPT**	**Weight percentage of the clay particles**
Bio_4	Temperature seasonality (SD * 100)	**CRVOL**	**Volumetric percentage of coarse fragments**
Bio_5	Max temperature of warmest month	**ORCDRC**	**Soil organic carbon content in permille**
Bio_6	Min temperature of coldest month	**PHIHOX**	**pH index measured in water solution**
**Bio_7**	**Temperature annual range**	**SLTPPT**	**Weight percentage of the silt particles**
**Bio_8**	**Mean temperature of wettest quarter**	SNDPPT	Weight percentage of the sand particles
**Bio_9**	**Mean temperature of driest quarter**	OCSTHA	Soil organic carbon stock
**Bio_10**	**Mean temperature of warmest quarter**		
Bio_11	Mean temperature of coldest quarter		
**Bio_12**	**Annual precipitation**		
Bio_13	Precipitation of wettest month		
**Bio_14**	**Precipitation of driest month**		
Bio_15	Precipitation seasonality (coefficient of variation)		
Bio_16	Precipitation of wettest quarter		
Bio_17	Precipitation of driest quarter		
Bio_18	Precipitation of warmest quarter		
Bio_19	Precipitation of coldest quarter		

#### Resolving multicollinearity among bioclimatic, topographic and soil variables.

**Figure 2.**
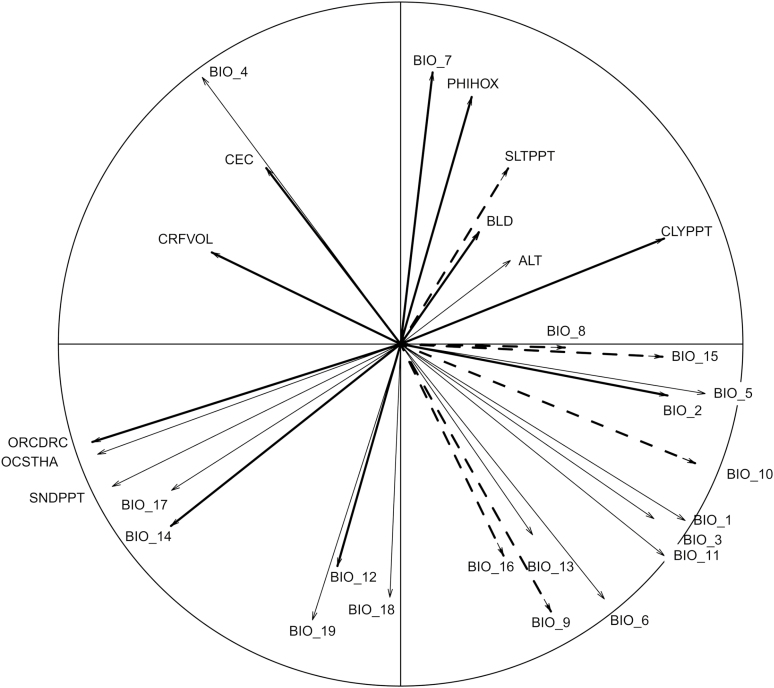
Correlation circle: PC1, the dimension with the most explained variance, is plotted on the horizontal axes; the second-most explanatory dimension is called PC2, plotted on the vertical axis. Inside this plot, the original climatic and soil variables are projected onto this 2D environment space. If two arrows are pointing in the same direction, then they are highly correlated. If they are orthogonal (at a 90° angle), they are unrelated and if they are pointing in opposite directions, they are negatively correlated. Indicated in bold arrows are the parameters that were used in the OLS models following the guidelines in our methods to avoid multicollinearity; indicated in dashed arrows are the parameters found to be significant in our models (Table 3).

In this study, abiotic factors based on species biology were not eliminated prior to building our models, as we have relatively little information on the influence of these specific abiotic parameters on breeding system traits. For example, though *T. perfoliata* is an early spring annual, it likely has an extensive seedbank. To visualize the relationships among all the potential abiotic factors (both climatic and soil), we performed a principal component analysis (PCA) which included all potential explanatory factors in our models (Fig. 2). A correlation matrix of standardized climatic variables, made in SDMtoolbox (Brown 2014), revealed multicollinearity among multiple climatic and soil variables within the sampling range **[see****Supporting Information—Appendix A****]**. Including multiple correlated variables violates assumptions of the model and provides no additional explanatory power. For instances of multicollinearity, we included only one variable of strongly correlated variables (using a conventional cut-off threshold of *R*^2^ > 0.70; Dormann *et al.* 2013; **see****Supporting Information—Appendix A**). In cases in which a variable was mathematically derived from another or multiple bioclimatic variables (i.e. temperature range), we included the non-derived variable (i.e. annual precipitation), as is standard practice (Guisan and Thuiller 2005; Guisan *et al.* 2007; Dormann *et al.* 2012). And in the remaining cases, we chose the variable that had the potential to most likely directly influence the breeding system of *T. perfoliata* using knowledge about the ecophysiology of this study system. For example, each soil parameter had measurements for two sampling depths and these measures were always highly correlated. We included the shallower sampling depth (10 cm) for each soil parameter because the roots of this species would be highly unlikely to reach 150 cm (Table 1; Fig. 2). And we chose the inclusion of temperature during the growing season over mean annual temperature. Our final models were run with nine climatic variables and seven soil parameters (Table 1; Fig. 2). Multicollinearity is problematic for biological interpretation of model results, so we exercise caution in extrapolating these results. In all cases in which a variable was significantly important in the OLS models, we discuss any correlation with other environmental variables in the results and discussion. The correlation matrix of all environmental variables within our sampling range is in the **Supporting Information—Appendix A**, and the relationships among all possible abiotic parameters can be seen visually in our PCA plot (Fig. 2).

#### OLS models.

We used OLS models in the Spatial Analyses in MacroEcology (SAM) program (Rangel *et al.* 2010) to test for associations between abiotic and breeding system variables. The global measurement of spatial autocorrelation among response variables (e.g. #CH) was not statistically significant (Wilcoxon signed-rank tests of Moran’s *I* values: *P*-value range = 0.62–0.92). However, because small-scale spatial patterns exist in most data sets, we included spatial covariates (e.g. latitude and longitude) as fixed effects.

All possible permutations were ranked by second-order Akaike information criteria (AICc), which corrects for a small sample size relative to possible model parameter predictors (*N* = 17 non-correlated climatic and soil variables; *N* = 65 535 permutations). We report the model with the lowest AICc score as per the AIC for model selection (Burnham and Anderson 2004).

## Results

### Variation in plant reproductive traits

Breeding system traits were highly variable across populations of *T. perfoliata* (Table 2; Fig. 1). Relative chasmogamy (pCH) was 0.23 ± 0.04 (*N* = 14; mean ± 1 SE; Table 2) among individuals per population, with CL flower production exceeding that of CH flower production in all but one highly CH population in Falkland, NC. Total flower production varied widely by population, with a coefficient of variation of 63 % and an overall average total flower production of 29.29 ± 4.98 (*N* = 14; mean ± 1 SE) flowers per individual. Total CH and CL flower production per individual was 7.10 ± 1.73 (*N* = 14; mean ± 1 SE) and 22.11 ± 3.76 (*N* = 14; mean ± 1 SE), respectively. Across all populations, there were 25 plants in which no CL flowers occurred, and 182 individuals with no CH flowers. All individual plants in our survey had at least one flower **[see****Supporting Information—File S1****]**.

**Table 2.** Fourteen study populations of *Triodanis perfoliata* along with geographic information and descriptive statistics of reproductive variables as means (±1 SE); total FL = total number of flowers, pCH = #CH/total flowers. Overall means (±1 SE) for each trait are given with the population-level coefficient of variation (CV).

Population	State	Latitude	Longitude	CH#	CL#	Total FL	pCH
Mason Farm	NC	35.88324	−79.01798	9.48 (1.45)	38.98 (3.38)	48.46 (4.38)	0.14 (0.02)
NY Botanic Gardens	NY	40.85984	−73.87558	2.36 (0.61)	28.98 (2.28)	31.34 (2.59)	0.057 (0.01)
Mahoney Property	NC	35.72059	−77.57575	8.37 (0.63)	6.814 (0.60)	15.19 (1.00)	0.58 (0.03)
GA Population 5026	GA	33.99055	−84.94263	5.28 (0.79)	23.56 (2.63)	28.84 (2.80)	0.18 (0.02)
Ivy Creek	VA	38.09029	−78.49368	6.39 (1.21)	26.75 (5.06)	33.14 (6.26)	0.18 (0.03)
Claytor	VA	37.38731	−79.53914	6.71 (0.96)	41.58 (5.18)	48.29 (5.93)	0.14 (0.01)
Calder	NY	41.12437	−73.73010	8.80 (1.79)	12.38 (1.47)	21.18 (1.36)	0.36 (0.06)
Red Bud Run	VA	39.19831	−78.12523	0.08 (0.09)	7.60 (0.35)	7.68 (0.41)	0.004 (0.004)
Meadowview	VA	38.14611	−77.38806	8.22 (0.91)	31.50 (2.89)	39.72 (3.43)	0.20 (0.02)
Breakneck Ridge	NY	41.44582	−73.97172	3.71 (0.90)	8.071 (1.38)	11.79 (1.66)	0.32 (0.07)
Baker 3084	AR	36.24252	−93.13755	4.10 (0.55)	8.40 (0.56)	12.50 (0.85)	0.29 (0.02)
Baker 3085	AR	36.24522	−93.13361	6.58 (0.93)	10.44 (0.93)	17.02 (1.41)	0.35 (0.04)
Bishop Farms	NJ	39.94000	−74.74596	27.31 (7.57)	47.75 (11.78)	75.06 (18.92)	0.31 (0.04)
Bull Shoals	MO	36.56721	−93.09628	2.06 (0.56)	16.74 (2.35)	19.81 (2.76)	0.10 (0.02)
							
Overall mean (SE)				7.10 (1.73)	22.11 (3.76)	29.29 (4.98)	0.23 (0.04)
CV				94	62	63	69

### OLS models

**Figure 3.**
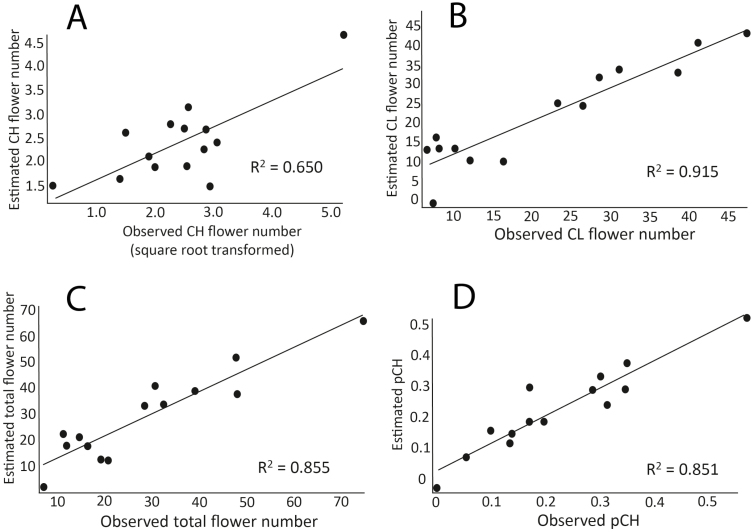
Model estimates versus observed reproductive trait measures in *Triodanis perfoliata* (*N* = 14); A-D refer to the four models in Table 3. Chasmogamous flower number is square-root transformed; pCH = #CH/total flowers.

**Figure 4.**
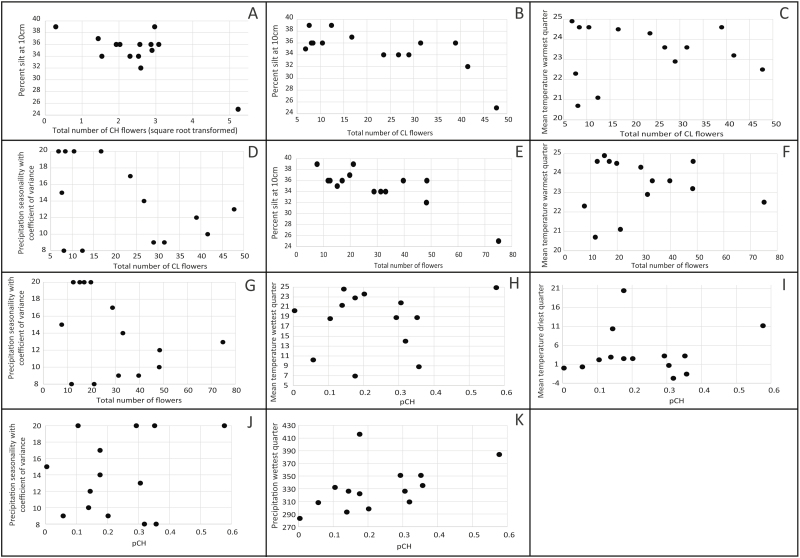
Scatter plots of 11 significant parameters (A-K) from OLS models against the observed breeding system trait for *N* = 14 populations of *Triodanis perfoliata*. Each full model and regression coefficients are described in Table 3.

Ordinary least squares models use the principle of least squares to choose the parameters of a linear function from a set of explanatory variables. Our models characterized a majority of the observed variation in the breeding system traits (range *R*^2^-value (min–max): 0.65–0.92; Table 3) with all breeding system traits tightly correlated to climatic and soil variables (Fig. 3). The three most influential abiotic variables in our models were the percentage of silt (10 cm sampling depth), precipitation seasonality (a coefficient of variation of monthly rainfall) and mean temperature of the warmest quarter (Table 3; Figs 3 and 4; **see****Supporting Information—File S2**).

**Table 3.** Regression coefficients and AICc scores from OLS minimum adequate models of reproductive response variables and abiotic parameters in *Triodanis perfoliata*. Reproductive traits include: number of CH flowers (CH#), number of CL flowers (CL#), total flowers (total FL) and #CH/total flowers (pCH). Explanatory parameters include: percent silt at measuring depth of 10 cm (% silt), mean temperatures ($\bar{x}$ temp.) at wettest, driest and warmest quarters, precipitation seasonality with coefficient of variation (precip. CV) and precipitation at wettest quarter.

	% silt	$\bar{x}$ temp. (wettest)	$\bar{x}$ temp. (driest)	$\bar{x}$ temp. (warmest)	Precip. CV	Precip. (wettest)	AICc	*R* ^2^
CH#	−0.233						40.06	0.65
CL#	−2.62			0.721	−2.604		103.2	0.86
Total FL	−4.11			0.670	−2.575		111.5	0.85
pCH		0.002	−0.003		−0.021	0.008	−15.5	0.92

The percentage of silt was significantly correlated (*R*^2^ > 0.70) with several other soil texture characteristics: percent sand content at two sampling depths, and percentage of soil silt at a 150 cm sampling depth, so these variables were excluded from OLS models **[see****Supporting Information—Appendix A****]**. Interestingly, the percentage of silt in the soil was the only explanatory variable in the best OLS model for CH#, indicating that soil characteristics alone can strongly predict average CH flower production in *T. perfoliata* populations (*R*^2^ = 0.65; Figs 3A and 4A). In our models, increasing silt content was associated with a decrease in CH#, CL# and total flower production (Figs 3 and 4A, B and E).

Unlike other climatic traits, precipitation seasonality did not correlate to any other climatic or soil parameters within our sampling range. In each of the models that included precipitation seasonality (CL#, total flowers, pCH), the regression coefficient was negative (Table 3), which suggests that increased variability in precipitation is associated with decreased flower production and lower relative chasmogamy (pCH) in *T. perfoliata* (Fig. 4D, G and J). Temperature during the warmest quarter (which is correlated with mean annual temperature) was included in two of our models. Increasing temperature is positively associated with CL# and total flower number in our models (Fig. 4C and F).

## Discussion

This study demonstrates that variation in reproductive traits is correlated to variation in environmental parameters across 14 widely distributed populations of *T. perfoliata* populations. In contrast to previous work in CL species, including *Triodanis*, we considered how long-term patterns in abiotic variation may be correlated with reproductive traits. Ecological studies at different spatial and temporal scales can detect different components of the genetic or plastic basis of traits such as cleistogamy. Our results expand upon previous work in *Triodanis* which used greenhouse experiments to examine how differences in soil and light conditions influence reproductive allocation (Colbert 2015), and a field study which examined the role of pollination in the plasticity of cleistogamy (Ansaldi *et al.* 2018). Colbert (2015) found that light increased the proportion of CH flowers, but soil did not influence reproductive plasticity. While these studies examined plastic changes in cleistogamy over the course of a single season, we found that environmental variation in climate and soil parameters (based on 30-year averages) explained between 65 and 92 % of reproductive trait variation within our sampling range. We did not examine variation in light availability across our populations, but in contrast to (Colbert 2015), soil characteristics were correlated with breeding system variation in our study. Our study emphasizes that environmental conditions are key drivers of breeding system variation. Specifically for dimorphic cleistogamy, our results are consistent with the hypothesis that resource limitation may favour the less expensive CL compared to CH flower type, and that this can be detected across large spatial scales.

Numerous studies have implicated the role of resource availability, specifically water, as an important factor in driving variation in CL species, with increased water availability generally favouring chasmogamy (Uphof 1938; Brown 1952; Langer and Wilson 1965; Jones *et al.* 2013). In our study, the relative production of CH flowers (pCH) was also positively associated with water availability (precipitation in the wettest quarter) among populations of *T. perfoliata*. Though it was not directly revealed in our best models, we hypothesize that water availability could also generally be associated with the total number of flowers a plant produces, with CH number highly correlated to total flower number. Gara and Muenchow (1990) suggested that the final CH flowers with relatively low seed set are subject to drier environments in *T. perfoliata*. Water stress can also increase levels of abscisic acid (Chrispeels and Varner 1966), and elevated abscisic acid triggers CL production (Lord 1981; Minter and Lord 1983). While low water availability is often implicated in reducing CH reproduction, several studies at smaller spatial scales have shown conflicting results. For example, soil moisture had no effect on reproductive resource allocation in *R*uellia *nudiflora* (Munguía-Rosas *et al.* 2012) nor *Viola striata* (Cortés-Palomec and Ballard 2006). And in *Viola lanceolata* (Violaceae), CL flower production was positively correlated to soil moisture, but there was no relationship between moisture and CH flower production (Ranua and Weinig 2010).

Soil texture is also known as an important factor in driving variation in reproductive traits, plant height, photosynthetic response and above-ground net primary production (Koenning *et al.* 1996; Epstein *et al.* 1997; Fravolini *et al.* 2005), but studies of the influence of soil texture on CL reproduction are sparse. Soil texture is an important determinant of water accessibility (Saxton and Rawls 2006). Soils with coarse texture (i.e. less silt) have larger pores, which increases saturated hydraulic conductivity, relative to finer, siltier soils (Jury and Flühler 1992). In other words, plants in soil with greater silt content likely experience lower water availability when soils are saturated with water (Jury and Flühler 1992). Wilken (1982) showed that increasing sand content resulted in lower soil saturation potentials and increased CH and CL flower numbers in *Collomia grandiflora*. We found a negative relationship between silt content of the soil and CL number, CH number and total flower production in *T. perfoliata*. These results conflict with a previous study in which soil properties influenced total flower production, but not the proportion of reproductive allocation in two populations *T. perfoliata* (Colbert 2015). Overall, our findings are consistent with the idea that water availability plays a major role in breeding system variation in this species. Of course, the percentage of silt in the soil is correlated to other soil traits (including sand and clay content), but our results do imply a general link between soil texture and allocation in the CL breeding system.

A number of papers emphasize the role of temperature as an environmental driver of CL breeding systems (Uphof 1938; Lord 1981). Several studies have shown that when threshold temperatures are reached, CH flowers are produced for the season (Uphof 1938; Lord 1981; de Clavijo and Jimenez 1993). Studies that experimentally manipulate temperature to assess reproductive allocation in cleistogamy response are not common, but results vary with no consistent pattern between annual and perennial life histories. For example, artificial warming treatments increased the proportion of CL fruit in *Viola praemorsa*, a perennial (Jones *et al.* 2013), while Langer and Wilson (1965) found no effect of temperature in *Bromus unioloides*, which can be annual or perennial. Our models on the annual *T. perfoliata* show temperature measurements can influence breeding system traits, but that the direction and magnitude of this effect varies depending on the reproductive trait and the other model parameters. We found that temperature is positively related to the proportional production of CH flowers in the wettest months, but negatively associated with this same trait during the driest months. Some of these patterns may occur because temperature influences other important parameters, such as water availability. For example, temperature-induced water stress can result in increased stomatal conductance and leaf respiration, and decreased leaf water potential under high temperatures (Erickson and Markhart 2001; Larcher 2003). Plants under high temperature stress can also experience bud abscission and flower sterility, which would also affect the number of flowers produced by a plant (Erickson and Markhart 2001).

Drawing general conclusions about the role of abiotic parameters in trait variation across CL species can be problematic. For example, soil moisture can interact with other factors that influence cleistogamy, such as pollination (Albert *et al.* 2011; Munguía-Rosas *et al.* 2012; Ansaldi *et al.* 2018). Additionally, the strength of response is likely to vary with plant life history, with annuals experiencing stronger selection than perennials on traits that maximize reproduction (Jasieniuk and Lechowicz 1987). Finally, genetic inheritance contributes to the observed variation in extent of chasmogamy (Clay 1982; Bell and Quinn 1987; Cheplick 2007). Our current study designed to detect patterns driven by long-term patterns in abiotic resources or cues. In comparison, studies on local scales can detect plastic responses that occur even within a single season (e.g. Ansaldi *et al.* 2018). At least in *T. perfoliata*, our current study combined with previous work (Colbert 2015; Ansaldi *et al.* 2018) suggests that breeding systems can be shaped by both long-term abiotic trends as well as stochastic factors that may change from season to season.

A high proportion of breeding system variation was explained by climatic and soil variables, including temperature and precipitation, suggesting that climatic change has the potential to influence breeding system allocation in *T. perfoliata*. For example, increasingly variable rainfall events (increased precipitation seasonality) are associated with lower relative CH, lower CL flowers and lower total flower production in this species. And, if temperatures across the range of this species increase, our models predict more CL flowers on *T. perfoliata*. Furthermore, the differences in magnitude and direction of responses based on abiotic factors suggest that impacts of climate change will vary by region. Overall, our models inform hypotheses about breeding system evolution, suggesting the possible environmental factors that could play key selective roles in shaping breeding system traits.

Our study emphasizes the general need to understand the influence of abiotic factors on plant breeding systems, and could have implications for understanding population responses to climate change. Specifically, water availability, soil texture and temperature appear to be key determinants of variation in reproductive traits in *T. perfoliata*. These findings empirically reinforce the theories that limited resource conditions are unfavourable for CH reproduction (Schoen and Lloyd 1984; Oakley *et al.* 2007), and expand upon previous work showing reproductive plasticity in this species (Goodwillie and Stewart 2013; Colbert 2015). Our models provide a framework for additional research about the influence of abiotic factors on CL breeding systems. In addition, a large proportion of the variation in reproductive traits in this species was explained by environmental factors (62–94 %). These results imply that changes in temperature and precipitation via climate change have the potential to influence plant breeding systems, and are congruent with a handful of other studies (Jones *et al.* 2013; Petry *et al.* 2016). Our work in *T. perfoliata* emphasizes the need to explore the impact of climate change in CL as well as other phenotypically plastic breeding systems.

## Sources of Funding

Funding for this research was provided via an NSF DEB-1142784 to S.J.F., and a GRFC from Southeast Missouri State University award to J.J.W.

## Contributions by the Authors

B.H.A., S.J.F., and J.J.W. posed the questions, designed the experiments and wrote the first drafts of the manuscript. B.H.A and J.J.W. performed analyses and revised the manuscript.

## Conflict of Interest

None declared.

## Supplementary Material

Supplementary S1

Supplementary S2

Supplementary Appendix A

## Supporting Information

The following additional information is available in the online version of this article—

**Appendix A.** A correlation matrix of standardized climatic variables, made in SDMtoolbox (Brown 2014).

**File S1.** A csv file of the reproductive traits measured in our study.

**File S2.** A csv file including the reproductive traits as well as the spatially explicit climate and soil variables used for producing the models in our study.
